# Naturally derived colloidal rods in microfluidic flows

**DOI:** 10.1063/5.0142867

**Published:** 2023-04-03

**Authors:** Vincenzo Calabrese, Amy Q. Shen, Simon J. Haward

**Affiliations:** Okinawa Institute of Science and Technology Graduate University, 1919-1 Tancha, Onna-son, Okinawa 904-0495, Japan

## Abstract

Naturally derived colloidal rods (CR) are promising building blocks for developing sustainable soft materials. Engineering new materials based on naturally derived CR requires an in-depth understanding of the structural dynamics and self-assembly of CR in dispersion under processing conditions. With the advancement of microfabrication techniques, many microfluidic platforms have been employed to study the structural dynamics of CR under flow. However, each microfluidic design has its *pros* and *cons* which need careful evaluation in order to fully meet the experimental goal and correctly interpret the data. We analyze recent results obtained from naturally derived CR and relevant rod-like macromolecules under microfluidic flows, with emphasis on the dynamical behavior in shear- and extensional-dominated flows. We highlight the key concepts required in order to assess and evaluate the results obtained from different CR and microfluidic platforms as a whole and to aid interconnections with neighboring fields. Finally, we identify and discuss areas of interest for future research directions.

## I. INTRODUCTION

**FIG. 1.**
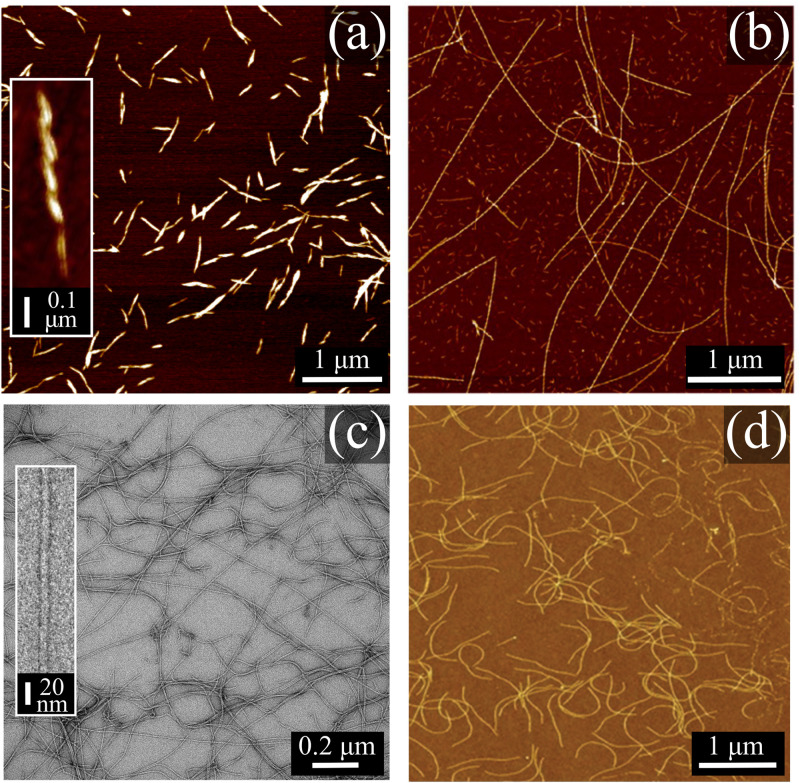
Images of commonly studied naturally derived CR. (a) Atomic force microscopy (AFM) image of cellulose nanocrystals (CNC) obtained in our laboratory as described previously.^9^ (b) AFM image of protein nanofibrils adapted from Jurado *et al.*, Biomacromolecules **22**, 2057–2066 (2021).^10^ Copyright 2021 Author(s), licensed under CC BY 4.0. (c) Pseudomonas phages, Pf1, negatively stained with 2% uranyl acetate aqueous solution imaged with a transmission electron microscope (previously unpublished data obtained by the authors). (d) fd viruses adapted from Lang *et al.*, Soft Matter **15**, 833–841 (2019).^11^ Copyright 2019 the Royal Society of Chemistry, licensed under CC BY 3.0.

Naturally derived colloidal rods (CR) are involved in a myriad of processes and applications. Well-known examples of naturally derived CR are cellulose nanocrystals [CNCs, shown in Fig. 1(a)] and cellulose nanofibers (CNFs), both of which are extracted from wood pulp and employed in fiber spinning and biomedical applications.^1,2^ Other types of carbohydrate-based CR can form *via* lateral aggregation of soluble carbohydrate chains in specific ionic environments, as reported for low acetylated gellan gum and carrageenan polysaccharides.^3,4^ Protein-based CR, referred to as protein nanofibrils or amyloids [Fig. 1(b)], are formed *via* self-assembly of hydrolyzed peptides under specific conditions of flow, pH, and ionic strength.^5,6^ Protein nanofibrils are of great interest due to their involvement in neurodegenerative diseases such as Alzheimer’s and Parkinson’s and their applications in food products (e.g., as thickening and gelling agents).^6–8^ Most naturally derived CR that can be produced in mass quantities for industrial purposes have a polydisperse contour length, $l_{c}$, (the length of the fully stretched CR) and a morphology that can significantly deviate from those of perfect rods [see CNC in Fig. 1(a)]. This implies that no available theory guarantees a reasonable prediction of structural and rheological properties of naturally derived CR dispersions under flow. Since these rod-like particles are suitable building blocks for the development of sustainable materials, establishing an experimental framework to study the dynamics of such CR under flow becomes essential. In contrast, filamentous viruses are a group of naturally produced CR with monodisperse $l_{c}$ and diameter, d, that can be adequately approximated by a cylindrical shape [see Pf1 in Fig. 1(c)]. This makes filamentous viruses particularly suited for testing theories and physics concepts regarding the flow behavior of rod-like particles.^11–16^ The most used viruses comprise tobacco mosaic virus (TMV), Pseudomonas phage (Pf1), fd [Fig. 1(d)], fdY21M, and M13k07.^17^ In contrast to single polymer chains in solutions, CR are relatively stiff due to the assembly of multiple macromolecules across the diameter. This supramolecular assembly across the CR diameter impedes CR from adopting different conformations arising from the rotational freedom around the backbone. Consequently, CR flexibility arises exclusively from thermal bending fluctuations along the backbone.^18^ CR flexibility is typically categorized according to their persistence length $l_{p}$, a material property related to the elastic modulus ( E) and the diameter ( d) of the CR, and the thermal energy $k_{b}T$ as $l_{p}\propto d^{4}E/k_{b}T$.^19^ Practically, the persistence length $l_{p}$ quantifies the length scale below which the CR behave as rigid rod-like segments. CR are commonly considered as rigid ( $l_{p}\gg l_{c}$) or semi-flexible ( $l_{p}∼l_{c}$). Compared to high molecular weight polymer chains in solutions, for which $l_{p}\ll l_{c}$, CR are considerably stiffer.

**FIG. 2.**
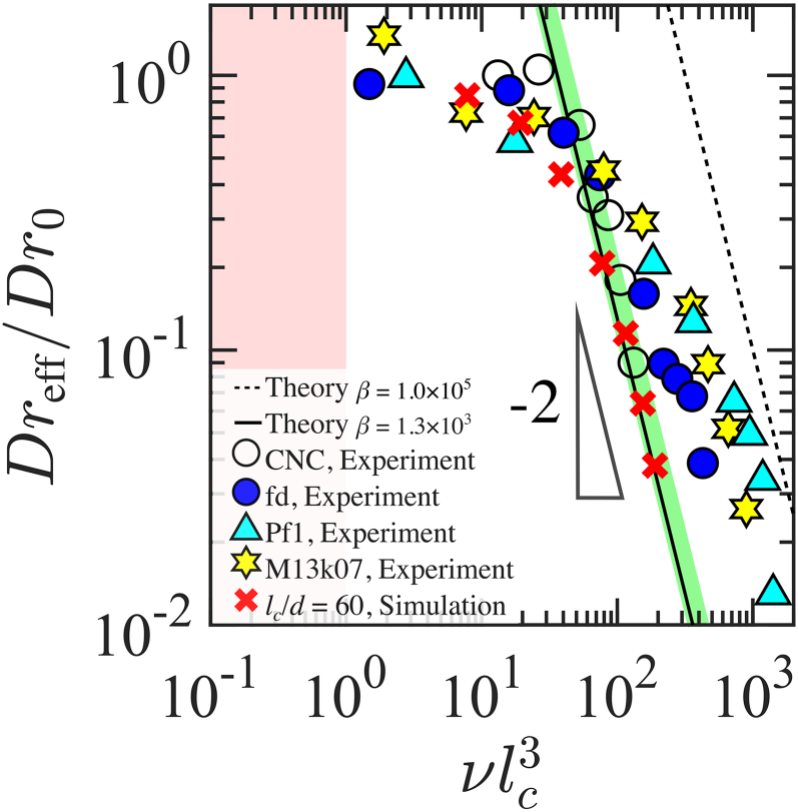
Normalized rotational diffusion coefficient ( $Dr_{\mathrm{eff}}/Dr_{0}$) as a function of $\nu l_{c}^{3}$ for cellulose nanocrystals, CNC [data obtained from Fig. 4(a) of Calabrese *et al.*, Macromolecules **55**, 5610–5620 (2022),^22^ re-plotted here with $\nu l_{c}^{3}$ as x-axis], fd and Pf1 viruses (data obtained from Barbé *et al.*, Phys. Fluids **32**, 053105 (2020).^23^ M13k07 viruses (data obtained from Abakumov *et al.*, Macromolecules **54**, 9609–9617 (2021)^24^ and numerical simulations for a rigid rod with aspect ratio, $l_{c}/d=60$ (data obtained from Tao *et al.*, J. Chem. Phys. **124**, 134906 (2006).^25^ The solid and dashed lines describe Eq. (2) with $\beta =1.3\times 10^{3}$ and $\beta =1.0\times 10^{5}$ (drawn as a reference), respectively. The green area represents the experimental boundaries of β found by Lang *et al.*^12^ for a library of monodisperse filamentous viruses. For fd and Pf1 viruses, $Dr_{0}$ is computed from Eq. (1). The colored area at $\nu l_{c}^{3}<1$ marks the dilute regime where $Dr_{\mathrm{eff}}/Dr_{0}=1$.

The concentration regimes for CR are based on the number of CR per unit volume, referred to as the number density $\nu =(4\phi_{v})/(d^{2}l_{c}\pi )$ (in $m^{-3}$), with $\phi_{v}$ being the volume fraction. CR are isolated from each other in the dilute regime when $\nu <l_{c}^{-3}$ and interacting in the semi-dilute regime, $l_{c}^{-3}<\nu <d^{-1}l_{c}^{-2}$.^20,21^ The effective rotational diffusion coefficient $Dr_{\mathrm{eff}}$ depends on the concentration regime. In the dilute regime, $Dr_{\mathrm{eff}}$ is given as^11,12,20^

$$Dr_{\mathrm{eff}}\equiv Dr_{0}=\frac{3k_{b}T\ln(l_{c}/d)}{\pi \eta_{s}l_{c}^{3}},$$ (1)

where $k_{b}=1.38\times 10^{-23}$ J/K is the Boltzmann constant, T is the absolute temperature, and $\eta_{s}$ is the solvent viscosity. By inspecting Eq. (1), $Dr_{0}$ is independent of ν, thus $Dr_{0}$ is predicted to be constant throughout the dilute regime, when interparticle interactions are negligible. In the semi-dilute regime, interparticle interactions lead to an effective rotational diffusion coefficient that decreases with increasing CR concentration (as shown in Fig. 2 for CNC,^22^ several filamentous viruses^23,24^ and compared with results from numerical simulations).^25^ According to the Doi–Edwards theory, in the semi-dilute regime,^20,26^

$$Dr_{\mathrm{eff}}\equiv Dr=\beta Dr_{0}{\left(\nu l_{c}^{3}\right)}^{-2},$$ (2)

where β is a dimensionless and length-independent prefactor ≫1 that governs the concentration range where the scaling $Dr/Dr_{0}\propto {\left(\nu l_{c}^{3}\right)}^{-2}$ is respected. From simulations and theory, it has been shown that $\beta =1.3\times 10^{3}$ (see simulation data and theory in Fig. 2).^20,25,27^ Recently, using filamentous viruses with distinct $l_{c}$, d, and $0.09<l_{c}/l_{p}<0.75$, it has been shown experimentally that $1.1\times 10^{3}<\beta <2.2\times 10^{3}$ (defined by the green area in Fig. 2), comparable to theoretical prediction.^12^ Nonetheless, deviations from the value of $\beta =1.3\times 10^{3}$ for ideal rigid rods have been reported.^8,28^ A deviation from the theoretical value can be linked to multiple factors. For instance, the selection of a single representative value for $l_{c}$ in a polydisperse CR population, the assessment of d and the density of the CR (affecting ν), interparticle attractive forces, and CR flexibility.

For $\nu >d^{-1}l_{c}^{-2}$, CR are in the concentrated regime and their diffusion becomes severely hindered as excluded volume interactions become important. In the concentrated regime, rigid rod-like particles can adopt a favorable orientation at rest, a phase that is referred to as the liquid crystal phase.^20,21^

The correct estimation of the effective rotational diffusion coefficient $Dr_{\mathrm{eff}}$ is important because it sets the value of the characteristic deformation rate E (i.e., $E\equiv \dot{\gamma }$ for the shear rate and $E\equiv \dot{\varepsilon }$ for the extensional rate, respectively) required to induce the alignment of the CR. Specifically, the Péclet number $Pe=|E|/Dr_{\mathrm{eff}}$ indicates the relative importance of the hydrodynamic forces over rotational Brownian diffusion. For Pe<1, colloidal rods are isotropically distributed via Brownian diffusion while for Pe≳1, hydrodynamic forces are sufficiently strong to favor alignment of the rods. Additionally, $Dr_{\mathrm{eff}}$ gives the relaxation time of the CR as $\tau =1/(6Dr_{\mathrm{eff}})$.^20^ The estimation of τ is pivotal for the synthesis of structurally ordered materials that rely on the flow-induced alignment of CR. For instance, to retain out-of-equilibrium CR orientations, physical or chemical processes able to lock the microstructure in place must occur within a time scale of ≪τ.

Microfluidic devices are versatile platforms to study the flow-induced alignment of CR in different flow fields and for the retrieval of relevant time- and length-scales associated with the CR. For instance, microfluidic devices can be designed to generate two-dimensional (2D) flows that provide, to a good approximation, a uniform flow through the channel height (e.g., microfluidics with rectangular cross sections and relatively high aspect ratio). 2D flows are convenient for flow visualization and for techniques aimed at probing the structural properties of the fluid, such as flow-induced birefringence (FIB) and small-angle scattering (SAS). Alternatively, three-dimensional (3D) flows, generated in microfluidic devices with symmetric and/or low aspect ratio cross-sections, are more representative of processing condition under which naturally derived CR may be employed (e.g., fiber spinning). Most importantly, microfluidics can be designed to generate not only shearing flows (the flow type generated by rotational rheometers) but also extensional flows and mixed flows comprising both shearing and extensional deformations. Thus, coupling microfluidics with flow visualization techniques, pressure sensors, FIB, and/or SAS techniques provides a comprehensive fingerprint of the structure–property relationship of colloidal rods in unique flow scenarios. Moreover, the small length scales adopted in microfluidic devices allow relatively high deformation rates while preserving creeping flow conditions.

This article provides an overview of microfluidic-based techniques to study the dynamics of naturally derived CR. Particular attention is given to CR in concentrations below the liquid crystal phase, where CR are isotropically distributed at rest but can adopt a favorable orientation under flow. In the following sections, we discuss some of the latest results regarding CR and relevant rod-like macromolecules under shearing and extensional flows.

## II. SHEARING FLOW

**FIG. 3.**
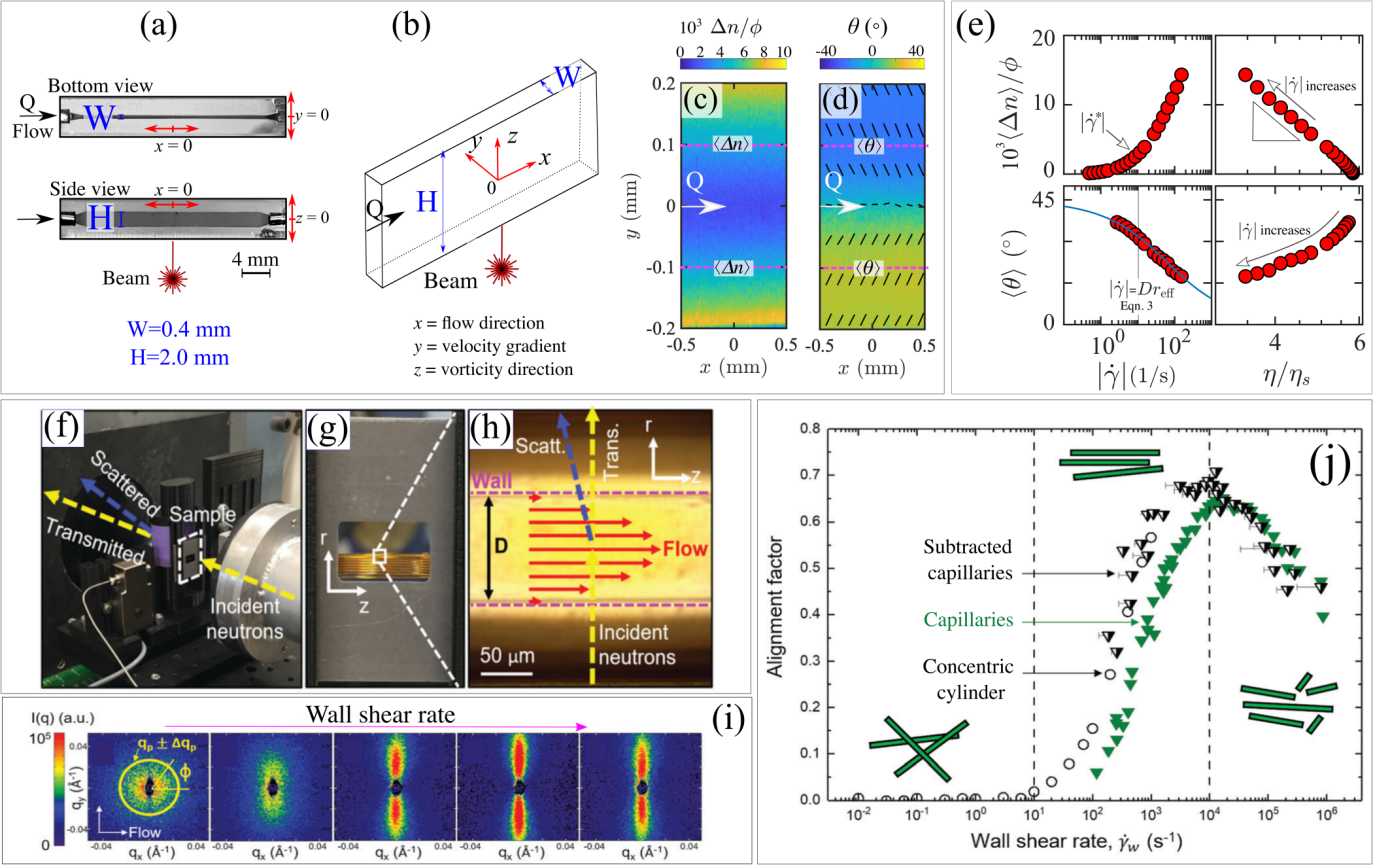
(a) Snapshots of a microfluidic platform with rectangular cross section from bottom and side views with (b) schematic drawing.^22^ (c) and (d) Time-averaged flow-induced birefringence (FIB) across the channel width for an aqueous CNC dispersion.^22^ (c) The normalized birefringence intensity ( Δn/ϕ) and (d) the orientation of the slow optical axis ( θ), given by the contour plot and depicted by superimposed solid segments to guide the eye. The horizontal dashed lines in (c) and (d) indicate locations where the spatially averaged birefringence, ⟨Δn⟩, and the spatially averaged angle of slow axis ⟨θ⟩ are obtained for a quantitative analysis. (e) ⟨Δn⟩ and ⟨θ⟩ as a function of $\left|\dot{\gamma }\right|$ ( $s^{-1}$) (left-side) and as a function of the steady shear viscosity normalized by the solvent viscosity, $\eta (\dot{\gamma })/\eta_{s}$ (right-side) for a 8 mg/ml CNC dispersion (semi-dilute). ⟨Δn⟩ and ⟨θ⟩ data as a function of $\left|\dot{\gamma }\right|$ ( $s^{-1}$) are obtained from Figs. 2(e) and 2(g) of Calabrese *et al.*,^22^ while the viscosity data as a function of $\left|\dot{\gamma }\right|$ are obtained from Fig. 5(a) of Calabrese *et al.*^22^ The black solid line in the bottom-left graph is the fitting to Eq. (3) and the vertical line indicates $|\dot{\gamma }|=Dr_{\mathrm{eff}}$ retrieved from Eq. (3). (f) Capillary RheoSANS (CRSANS) sample environment with (g) a zoom-in at the capillary coil and (h) the fused silica capillary.^29^ (i) Typical 2D SANS patterns for a worm-like micellar solution upon increasing shear rate (from left to right). (j) Alignment factors ( AF) obtained from CRSANS for a worm-like micellar solution. AF are compared for the capillary geometry (green triangles) against the isolated high shear region referred to as subtracted capillaries (half filled triangles) and the concentric cylinder geometry (open circles) from standard rheo-SANS experiments. Images in (a)–(d) are adapted from Calabrese *et al.*, Macromolecules **55**, 5610–5620 (2022).^22^ Copyright 2022 Author(s), licensed under CC BY-NC-ND 4.0. Images in (f)–(j) are adapted from Murphy *et al.*, Soft Matter **16**, 6285–6293 (2020).^29^ Copyright 2020 the Royal Society of Chemistry.

Understanding the flow-induced alignment of rod-like particles is pivotal for developing materials with structural anisotropy. Although mixed flows are present in real-life applications, studying the shear-induced alignment of CR can provide insightful information that can be interpreted and analyzed based on existing literature and be directly correlated with established rheological parameters (e.g., steady shear viscosity and steady shear stress). In our research group, we have used glass microfluidic devices, fabricated by selective laser-induced etching, with high aspect ratio [ H/W=5, where H=2mm and W=0.4mm are the channel height and width, respectively, Figs. 3(a) and 3(b)] to generate good approximations of 2D purely shearing flows.^30,31^ Using a straight microfluidic device with a rectangular cross section [Figs. 3(a) and 3(b)], we have probed the alignment of CR such as CNC, worm-like block copolymers, and protein nanofibrils in the flow-velocity gradient plane using FIB.^9,22,32–34^ FIB provides two parameters related to the structural anisotropy of the fluid, namely, the birefringence, Δn, and the orientation of the slow optical axis, θ. For randomly oriented CR dispersed in an isotropic medium (e.g., water), Δn=0, while flow-induced alignment of CR is marked by Δn>0. CR are usually optically positive, so that the orientation of the slow optical axis, θ, captures the direction of orientation of the rods.^35^ For each volumetric flow rate ( Q, m ${}^{3}$/s), FIB provides the spatial distribution of the birefringence, Δn, [shown in the normalized form as Δn/ϕ, where ϕ is the mass fraction of the CR, in Fig. 3(c)] and the orientation of the slow optical axis, θ [Fig. 3(d)]. Analogous mapping of the flow-induced orientation of CR can be retrieved using scanning-small-angle x-ray scattering, referred to as scanning-SAXS.^36–39^ For scanning-SAXS, each scattering pattern is acquired along the region of interest point by point, making typical acquisition times long compared to FIB. For instance, for a dispersion of protein nanofibers, scanning-SAXS required an acquisition time of ∼5 min to scan 1 ${\mathrm{mm}}^{2}$ for a single flow rate, while at most a few seconds would be required for a typical FIB experiment.^36^ Importantly, the birefringence, Δn, and the order parameter, S, obtained from small-angle scattering techniques (computed from the anisotropy of the 2D scattering patterns) provide comparable information of the CR orientation under flow as Δn∝S.^40–42^ On the other hand, compared to FIB, SAS can provide additional information regarding the CR morphology and particle–particle interactions within the same experiment when performed on the sample at rest. For quantitative comparisons of Δn and θ, it is convenient to spatially average these quantities (denoted as ⟨Δn⟩ and ⟨θ⟩) across a location of the microfluidic device where the deformation rate is constant and to plot them as a function of the respective deformation rate. For instance, in our previous publications using the microfluidic design given in Figs. 3(a) and 3(b), at each flow rate, we averaged Δn along a section of the microchannel located at y=±0.1 mm [see dashed lines sketched in Figs. 3(c) and 3(d)].^9,22,33^ These specific locations were chosen because they correspond to the middle distance between the centerline ( y=0 mm) and the channel wall, thus in between the location of the lowest and highest shear rate, respectively. Once the deformation rate at specific channel location is obtained [e.g., experimentally using micro-particle image velocimetry ( μ-PIV) or obtained computationally by choosing a suitable rheological model], the spatially averaged birefringence, ⟨Δn⟩, and the spatially averaged slow orientation angle, ⟨θ⟩, vs $\left|\dot{\gamma }\right|$ can be constructed, as shown for a semi-dilute CNC dispersion [left-side graphs in Fig. 3(e)]. Since the birefringence, Δn, is directly linked to the number of aligned CR segments along the optical path, the critical shear rate at the onset of birefringence ( $\left|{\dot{\gamma }}^{*}\right|$) provides a good estimation of $Dr_{\mathrm{eff}}$ since $|{\dot{\gamma }}^{*}|=Dr_{\mathrm{eff}}$ (i.e., Pe=1). For a shear flow, the orientation angle of the CR with respect to the flow direction in the flow velocity-gradient plane can be generally described as

$$\langle \theta \rangle =\frac{\pi }{4}-\frac{1}{2}\arctan\left[{\left(\frac{\left|\dot{\gamma }\right|}{6Dr_{\mathrm{eff}}}\right)}^{\alpha }\right],$$ (3)

where 0<α≤1 is a stretching exponent that accounts for particle polydispersity.^43–46^ According to Eq. (3), at low $\dot{\gamma }$ ( Pe→0), the CR orientation $\langle \theta \rangle \to 45^{{}^{\circ}}$, while at high $\dot{\gamma }$ ( Pe→∞), $\langle \theta \rangle \to 0^{{}^{\circ}}$.^47^ By fitting Eq. (3) to experimental data, it is possible to have a second estimate of $Dr_{\mathrm{eff}}$ to compare with that obtained by the onset of birefringence using the criteria $|{\dot{\gamma }}^{*}|=Dr_{\mathrm{eff}}$. For the exemplary CNC data, the trend of ⟨θ⟩ as a function of $\left|\dot{\gamma }\right|$ is well captured by Eq. (3) using α=0.39 and $Dr_{\mathrm{eff}}\approx 10s^{-1}$ [bottom-left graph in Fig. 3(e)]. At $|\dot{\gamma }|=|{\dot{\gamma }}^{*}|=Dr_{\mathrm{eff}}=10s^{-1}$ (i.e., Pe=1), the birefringence increases significantly, indicating that the two experimental approaches to obtain $Dr_{\mathrm{eff}}$, based on the onset of birefringence and the orientation of the slow optical axis [Eq. (3)], are in close agreement with each other [left-side graphs in Fig. 3(e)].

With relative ease, it is possible to link the flow-dependent structural properties of the fluid, such as ⟨Δn⟩ and ⟨θ⟩ as a function of $\left|\dot{\gamma }\right|$, with the respective rheological properties obtained from rotational rheometry. As an example, using the published data for a semi-dilute CNC dispersion, we plot ⟨Δn⟩ and ⟨θ⟩ a as a function of the normalized shear viscosity, $\eta /\eta_{s}$ [right-side graphs in Fig. 3(e)] at matching values of $\left|\dot{\gamma }\right|$.^22^ These plots are instructive because they highlight the connection between the CR alignment and the rheological property. For CNC, the shear viscosity decreases exponentially with the increasing ⟨Δn⟩ as $\eta /\eta_{s}\propto e^{-A\langle \Delta n\rangle /\phi }$, with A being an empirical fitting parameter, following a straight line in a semi-log representation [note $\eta /\eta_{s}$ is given in log scale, right-side graphs in Fig. 3(e)]. A similar exponential relation between the CR alignment and shear viscosity has been shown for fd-viruses^48^ and other worm-like nanoparticles.^33,49^ On the basis of the limited experimental data available, it is unclear whether such an exponential relationship between Δn (or S) and η can be treated as a universal feature of CR under shearing flow (especially for polydisperse CR),^50^ leaving interesting opportunities for further investigation. The link between the CR alignment and the resulting rheological properties can also be drawn by analyzing $\eta /\eta_{s}$ as a function of projected particle orientation in the flow-velocity gradient plane, given by ⟨θ⟩ [bottom-right graph in Fig. 3(e)]. The link between the CR alignment and standard rheological parameters underpins the physical origin of the flow behaviour of CR dispersions and enables detailed testing of microstructural constitutive models.

With typical 2D microfluidic geometries, such as the one shown in Figs. 3(a) and 3(b), maximum shear rates of $O(10^{3})s^{-1}$ can be achieved while ensuring the creeping flow of low viscosity aqueous fluid samples. Using capillary rheo-small-angle neutron scattering (CRSANS), the steady shear viscosity and alignment of fd-viruses and worm-like micelles have been studied simultaneously for shear rates up to $10^{6}s^{-1}$.^29,51,52^ Although worm-like micelles are not colloidal rods, they have an elongated shape and have been used as model fluid for validating the working principle of CRSANS. CRSANS combines neutron scattering with pressure drop measurements to provide, at once, both the shear viscosity and a measure of macromolecular conformations under flow. CRSANS uses a capillary with a typical inner diameter D∼0.1 mm coiled in circular loops to increase the scattering volume located within the neutron beam path [Figs. 3(f), 3(g), and 3(h)]. Murphy *et al.*^29^ compared the alignment factor ( 0≤AF≤1) computed from the anisotropy of the 2D scattering patterns of a worm-like micellar solution [shown in Fig. 3(i)] using CRSANS and traditional rheoSANS in a concentric cylinder geometry. The authors took particular care at isolating the scattering contribution near the walls of the capillary from the large span of shear rate experienced by the microstructure through the capillary cross section. As such, the AF obtained from the full geometry [green triangles in Fig. 3(j)] could be compared with the AF obtained after isolation of the scattering contribution at the capillary wall [half filled triangles in Fig. 3(j)]. The AF at the capillary wall was found to be in good agreement with the one obtained from conventional rheoSANS performed with a concentric cylinder geometry [open circles in Fig. 3(j)], validating the isolation of the scattering contribution at the capillary wall. Interestingly, by using CRSANS, Murphy *et al.* could probe a decreasing value of AF for $\dot{\gamma }\geq 10^{4}s^{-1}$ associated with the physical breakage of the worm-like micelles that could not be accessed by using conventional rheoSANS. The recent successful application of CRSANS to the study of fd-virus^51^ indicates that the technique will be useful for probing the shear-induced alignment of CR. In particular, we envision that CRSANS will be especially useful to probe the shear-induced alignment of CR with relatively high $Dr_{\mathrm{eff}}$ (e.g., relatively small CR in low viscosity solvents) that require high shear rates to align significantly.

## III. EXTENSIONAL FLOW

**FIG. 4.**
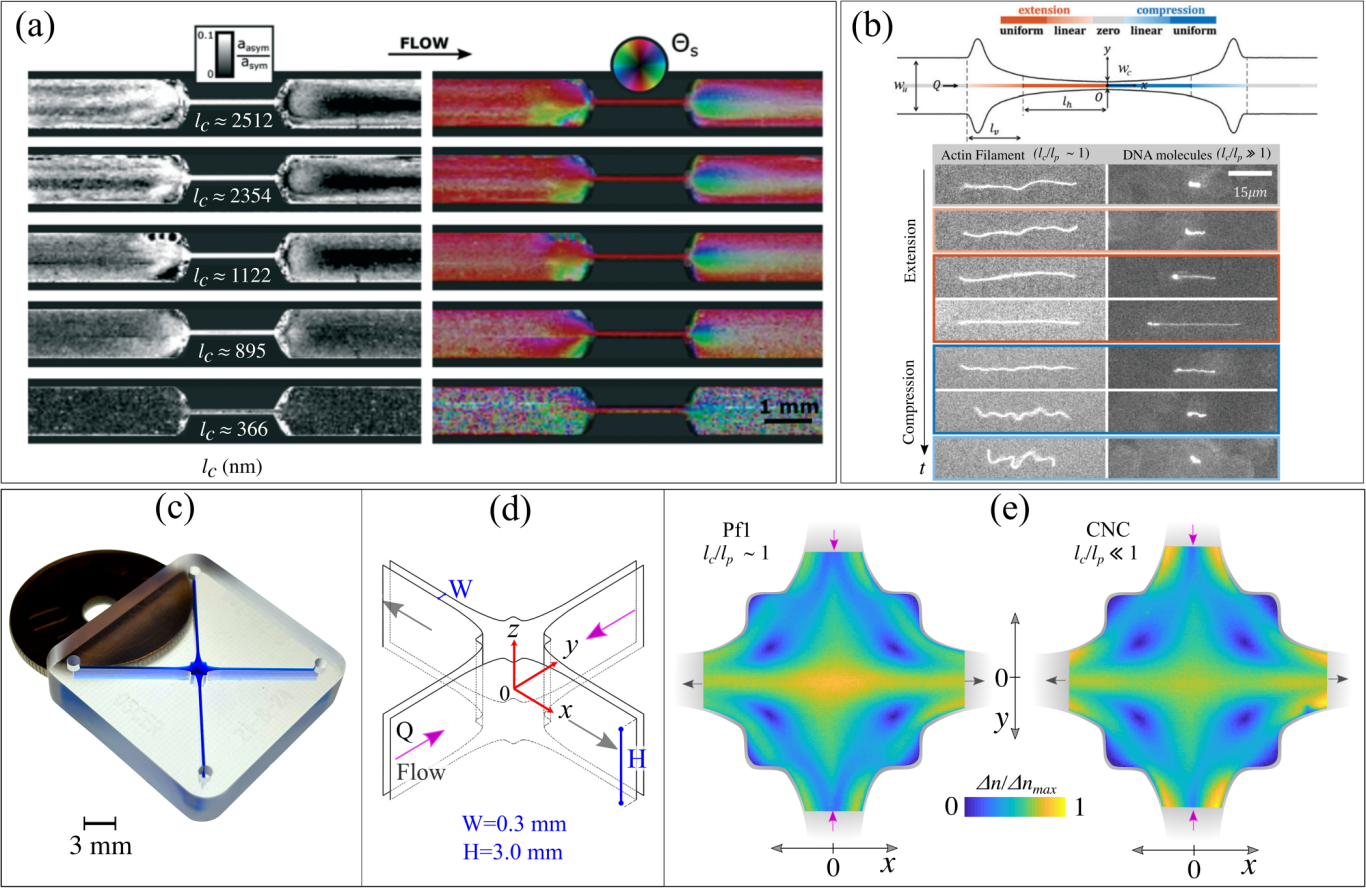
(a) Protein nanofibril dispersions in a tapered contraction-expansion geometry flowing at a constant flow rate probed by scanning-SAXS.^36^ Protein nanofibrils with increasing average contour lengths ( $l_{c}$) from bottom to top. The parameter $a_{\mathrm{asym}}/a_{\mathrm{sym}}$ indicates the extent of protein nanofibril alignment while the particle orientation is given by the direction perpendicular to $\Theta_{S}$. (b) Sketch of the optimized hyperbolic microchannel.^53^ The colored strand at the channel centerline maps the region with zero, uniform, and homogeneous extension rate. Images at the bottom of panel (b) show a series of snapshots for an actin filament and DNA flowing through the extension and compression part of the optimized geometry. (c) Optimized shape cross-slot extensional rheometer (OSCER) analogous in shape to the one used in previous publications (previously unpublished photograph obtained by the authors).^9,33,54,55^ (d) Schematic drawing of the OSCER device in (c). (e) Typical birefringence patterns in the OSCER device given in (c) for an aqueous dispersion of CNC (at 1 mg/ml, dilute regime), prepared as indicated previously,^9^ and a relatively more flexible Pf1 dispersed in Tris-EDTA buffer at *p*H 8 (at 0.1 mg/ml, semi-dilute regime) at comparable values of Pe∼2. The CNC is sourced from Celluforce (Canada) and the Pf1 from ASLA Biotech (Latvia). FIB was measured using a high-speed polarization camera (CRYSTA PI-1P, Photron Ltd, Japan) installed on a home-built upright microscope (previously unpublished data obtained by the authors). Image in (a) is adapted from Lutz-Bueno *et al.*, Lab Chip **16**, 4028–4035 (2016).^36^ Copyright 2016 the Royal Society of Chemistry. Image in (b) is adapted from Liu *et al.*, Soft Matter **16**, 9844–9856 (2020).^53^ Copyright 2020 the Royal Society of Chemistry. Image in (d) is adapted from Calabrese *et al.*, Macromolecules **55**, 10031–10042 (2022).^33^ Copyright 2022 Author(s), licensed under CC BY-NC-ND 4.0.

Extensional flows are ubiquitous in most processing conditions, such as in jetting, dispensing and spraying of formulations, fiber spinning, and emulsion formation. A convenient way to study CR dynamics in extensional-dominated flows is by using microfluidics geometries. CR dynamics in planar extensional-dominated flows have been mainly studied using contraction-expansion (expansion-contraction)^36,56–58^ and cross-slots geometries.^9,33,38^ The advantage of contraction-expansion geometries is that they require the control of a single stream, making them relatively simple to operate. Abrupt contraction-expansion geometries cause a sharp velocity change around contraction and expansion throats, therefore an inhomogeneous extension rate, $\dot{\varepsilon }$, along the centerline. Tapered contraction-expansion or hyperbolic-shaped geometries do not improve significantly the development of a homogeneous extension rate, but on the positive side, the absence of sharp corners minimizes the chance of entrapment of residual air bubbles.^53^ An example of contraction-expansion geometry with a tapered design is given in Fig. 4(a), used to study via scanning-SAXS the flow-induced alignment of distinct protein nanofibrils with varying average $l_{c}$.^36^ From the anisotropy of 2D scattering patterns, the authors retrieved a relative measure of the CR alignment ( $a_{\mathrm{asym}}/a_{\mathrm{sym}}$), qualitatively analogous to the alignment factor ( AF) and the order parameter S discussed above. Specifically, for $a_{\mathrm{asym}}/a_{\mathrm{sym}}>0$, the CR adopt a favorable orientation. For a given flow rate, longer protein nanofibrils are more likely to undergo flow-induced alignment than shorter ones; thus, $a_{\mathrm{asym}}/a_{\mathrm{sym}}$ generally increases with increasing $l_{c}$ [from bottom to top in Fig. 4(a)]. Practically, for the same flow rate and same particle concentration, the longer protein nanofibrils experience a higher Pe than shorter ones. Additionally, the highest extent of alignment is observed in the areas where high deformation rates, both $\dot{\gamma }$ (near the channel walls) and $\dot{\varepsilon }$ (at the contraction and expansion throats), are experienced by the CR. A more comprehensive vision of the flow-induced alignment of CR is gained by mapping the orientation angle of anisotropic scattering patterns $\Theta_{S}$ shown in the right-hand panel of Fig. 4(a), where $\Theta_{S}$ is perpendicular to the CR orientation in real space.

When the primary interest is the study of CR in extensional flows, the simple designs of contraction-expansion geometries discussed above may not be the most suited, primarily due to the inhomogeneity of the extension rate. Indeed, CR flowing in such devices encounter a complex flow with constantly varying $\dot{\varepsilon }$ that is transient in the *Lagrangian* frame (i.e., from the fluid point of view) although the flow is steady in the *Eulerian* frame (i.e., the flow is steady in time). This makes data interpretation difficult, raising questions such as “what is the relevant extension rate and time-scale at play?,” “what is the effect of the inhomogeneous extension rate?,” and “has the fluid accumulated enough strain to align the CR?,” the answers to which are not always straightforward.

To generate shear-free flows with extension rates that are constant over a sufficiently large area, specific microfluidic geometries are required. For instance, the optimized hyperbolic microchannel device as shown in Fig. 4(b) has been designed to generate a relatively large length where uniform planar extension and compression rates are generated along the centerline of the device. As a proof of concept, the authors tested the device to track the dynamics of fluorescently labeled actin filaments ( $l_{c}/l_{p}∼1.6$) and a more flexible DNA ( $l_{c}/l_{p}∼1200$). The actin filament displayed the suppression of bending fluctuations in the extension zone and a buckling instability in the compression zone.^53,59^ Conversely, the DNA showed a sharp coil-to-stretch transition in the extension zone and a stretch-to-coil transition in the compression zone.^53^ In principle, this device can be easily coupled with other techniques (such as FIB or SAS measurements) to probe the average orientation of the CR in a homogeneous extensional flow. A similarly shaped microfluidic geometry to that shown in Fig. 4(b) has also been designed to generate homogeneous uniaxial extensional flows.^60^

In our laboratory, we have used an optimized version of the cross-slot device, referred to as the optimized shape cross-slot extensional rheometer (OSCER), to study the CR alignment in planar extensional flows [Figs. 4(c) and 4(d)].^9,32,33^ The OSCER has two incoming and two outgoing flows placed orthogonal to each other and a high aspect ratio [ H/W=10, with H=3mm and W=0.3mm for the device in Figs. 4(c) and 4(d)] producing a good approximation to the 2D flow.^54,61^ The OSCER generates a stagnation point at the center of symmetry of the geometry (i.e., x=y=0). Thus, fluid elements traveling through the stagnation point have a long residence time and a large accumulated strain. Additionally, the OSCER shape is numerically optimized to generate a constant and shear-free, extensional deformation around the stagnation point. These features make the OSCER geometry ideal for studying the CR alignment in nearly perfect planar extensional flows that are steady in both *Lagrangian* and *Eulerian* frames. In Fig. 4(e), we provide typical flow-induced birefringence patterns obtained in the OSCER for two distinct CR dispersions at matched Pe∼2. Interestingly, the bacteriophage Pf1 ( $l_{c}/l_{p}∼1$) and a more rigid CNC ( $l_{c}/l_{p}\ll 1$) display different birefringence fingerprints. The rigid CNC displays the highest birefringence in shear-dominated regions near the walls of the inlets and outlets. Contrarily, the semi-flexible Pf1 displays the highest birefringence along the extensional axis (i.e., along the x-axis at y=0 mm) where the flow is purely extensional. This highlights the fact that extensional forces are significantly more effective at stretching and aligning semi-flexible CR than shearing forces. Indeed, for structures more flexible than Pf1 such as a 1.6 MDa hyaluronic acid (polyelectrolyte in solution) with $l_{c}/l_{p}∼$250, it has been shown that the birefringence is mainly localized as a strand along the extensional axis.^55^ This indicates that extensional deformations become a requirement to induce stretching and alignment of flexible particles. Odell and Keller^62,63^ originally pointed out the different birefringence patterns produced in cross-slot devices and four-roll mills between flexible and relatively rigid rod-like macromolecules. The extent of localization of birefringence around the stagnation point was referred to as the “localization of the orientation effect.” The physical interpretation of this phenomenon is that stiff rod-like macromolecules such as CNC require only a small amount of strain to align. Consequently, fluid elements traveling far from the stagnation point can accumulate enough strain to align the CR and give rise to birefringence. Contrarily, more flexible macromolecules such as the Pf1 require a higher strain to stretch and align. Thus, only the fluid elements that pass close to stagnation will accumulate enough strain to orient the Pf1 segments, leading to the localization of the birefringence along the extensional axis. A quantitative understanding of localization of the orientation effect could potentially serve as a methodology to retrieve a statistically robust measure of the particle flexibility that is model-free.

**FIG. 5.**
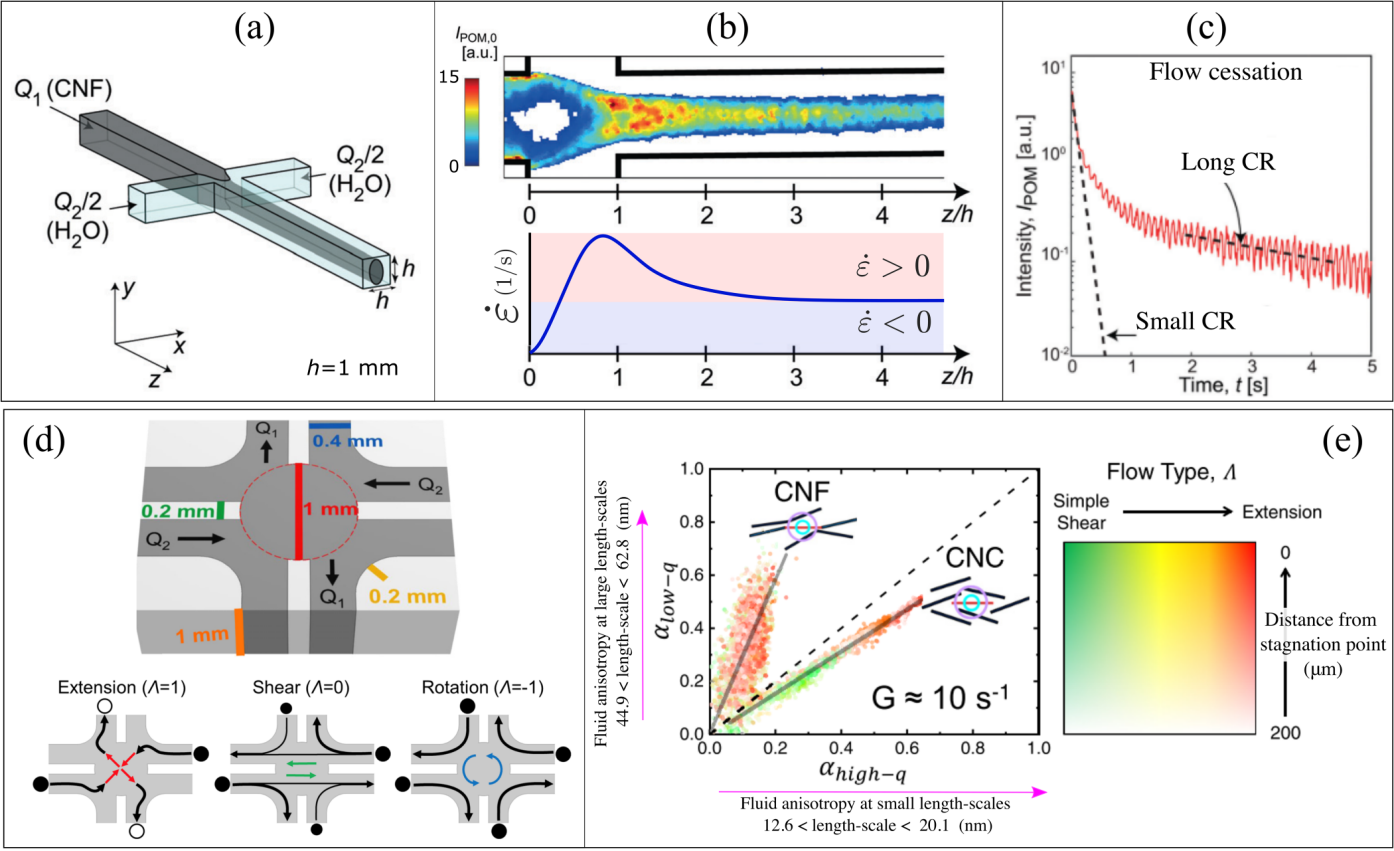
(a) Schematic of the flow-focusing (FF) device and (b) example of a polarized optical microscopy image during the steady flow of a cellulose nanofibrils (CNF) dispersion with respective sketch of $\dot{\varepsilon }$ along the centerline. $I_{\mathrm{POM}}$ is the light intensity passing through the sample sandwiched between two crossed polarizers and expected to be proportional to the square of the birefirngence ( $I_{\mathrm{POM}}\propto \Delta n^{2}$) in the given experimental conditions. (c) Typical flow cessation experiment for an aqueous CNF dispersion.^56^ The $I_{\mathrm{POM}}$ decays, from an initial steady state, after flow stoppage at t=0 (s). (d) Sketch of the fluidic four-roll mill (FFoRM) with the operational scheme for generating extensional, shearing, and rotational flows.^64^ (e) Comparison of the extent of alignment of CNC and CNF dispersions flowing in the FFoRM as probed by scanning-SAXS.^37^ The CR dispersions are compared at a nearly constant deformation rate while varying flow type from the shearing flow (green data points, Λ=0) to extensional flow (red data points, Λ=1). The color saturation of the data points indicates the proximity to the center of the device where the measurement was made. Relatively high color saturation indicates close proximity to the stagnation point and relatively high accumulated strain. The CR alignment is probed at two distinct length scales. A length scale that contains information of the particle alignment ( $\alpha_{\mathrm{high}-q}$), and a length scale that also probes the spatial distribution of the neighboring particles ( $\alpha_{\mathrm{low}-q}$). Images in (a)–(c) are adapted from Rosén *et al.*, Soft Matter **16**, 5439–5449 (2020).^56^ Copyright 2020 the Royal Society of Chemistry, licensed under CC BY 3.0. Image in (d) is reproduced from Corona *et al.*, Sci. Rep. **8**, 15559 (2018).^64^ Copyright 2018 Author(s), licensed under CC BY 4.0. Image in (e) is adapted from Corona *et al.*, Phys. Rev. Mater. **6**, 114 (2022).^37^ Copyright 2022 the American Physical Society.

The flow-focusing (FF) microfluidic geometry [sketched in Fig. 5(a)] has been used to study the dynamics of CNC and CNF dispersions in conditions relevant to fiber spinning.^39,65,66^ The FF device requires the control of three streams, the core stream containing the CR dispersion and two sheath streams containing a secondary fluid that focuses the core flow toward the centerline of the outlet channel [Fig. 5(a)]. Contrarily to the planar extension generated by the devices discussed above, the FF geometry produces uniaxial extension around the center axis of the outlet channel. The magnitude of the volumetric flow rate at the core stream ( $Q_{1}$) and the ratio $Q_{1}/Q_{2}$ dictate the extension rate.^67^ A point of commonality with non-optimized contraction–expansion geometries is the inhomogeneous extension rate, $\dot{\varepsilon }$, encountered by the CR traveling along the centerline where $\dot{\varepsilon }$ peaks around the center of symmetry of the geometry and quickly decreases to zero [Fig. 5(b)]. An effect of this transient $\dot{\varepsilon }$ experienced by the CR can be understood by following an hypothetical CR traveling along the centerline of the FF geometry. This CR requires some time to adopt the new orientation dictated by the encountered deformation rate. Consequently, the change in birefringence along the centerline does not occur instantaneously with varying $\dot{\varepsilon }$. This is evident downstream of the focusing point [ z/h>3, Fig. 5(b)] where the birefringence at the channel centerline persists although $\dot{\varepsilon }=0$. CR flexibility and interparticle interactions are expected to drastically affect the time required for a CR to adopt its favorable orientation under the flow.

The FF geometry has been used to estimate $Dr_{\mathrm{eff}}$ using the flow cessation method.^28,56,68^ In a classic flow cessation experiment, CR dispersion is brought to a constant and steady flow rate such that most particles align with the flow (i.e., Pe≫1).^8,43,62,69,70^ Subsequently, the flow is suddenly stopped and the birefringence decay is monitored over time ( t) [Fig. 5(c)]. In the context of polydisperse CR, two distinct decays of birefringence have often been observed in a semi-logarithmic representation [e.g., in Fig. 5(c)].^28,56,68^ The presence of two distinct birefringence decays have been associated with the relaxation process of two dominant size populations (as indicated by dashed lines in Fig. 5).^56^ Small rods relaxing fast, at small time-scales, while long rods relaxing at longer time-scale.^56^ Alternatively, the decay of birefringence has been described in terms of a continuous distribution of relaxation times related to the spectra of particle sizes.^28,43,68^ The decay of the birefringence is captured by an exponential decay,

$$\Delta n/\Delta n_{0}=e^{{\left(-t6Dr_{\mathrm{eff}}\right)}^{\alpha^{\prime }}},$$ (4)

where $\Delta n_{0}$ is the steady-state birefringence before flow stoppage and $0<\alpha^{\prime }\leq 1$ is a stretching exponent that captures particle polydispersity.^43,69^ For a single relaxation mechanism $\alpha^{\prime }=1$.^71^ The flow cessation experiment is versatile as it can be used to estimate $Dr_{\mathrm{eff}}$ using, in principle, any microfluidic or rheometric flow. Another interpretation of the flow cessation experiment for monodisperse CR in the semi-dilute regime is based on the concept of *tube dilation* from the Doi Edwards theory.^20,26^ In the Doi and Edwards vision, the rods can be described as confined in a imaginary tube. The tube diameter represents the average distance that the rods can move perpendicularly to their main axis unconstrained by neighboring rods.^20^ As the rods orient under flow, the confinement exerted by the surrounding rods minimizes and the tube dilates. With this in mind, upon flow stoppage, the relaxation process at short time scales is expected to be similar to that occurring in the dilute regime as the CR are unconstrained at short time-scales. Contrarily, as the tube contracts at longer time scales, the rods again perceive the surrounding rods, thus slowing down the return to isotropy. Based on this interpretation, two distinct exponential decays are expected in the semi-dilute regime. A fast relaxation process at short-time scale corresponding to $Dr_{0}$ and a slower relaxation process at longer times corresponding to Dr. Early experiments on stiff polymers have supported this latest concept.^62^ However, it is unclear to what extent polydispersity plays a role in this latest interpretation of the flow cessation experiment.

Recently Corona *et al.*^37,64^ have examined CR dynamics in the fluidic four-roll mill (FFoRM), originally introduced by Lee *et al.*^72^ The FFoRM is composed of eight channels, capable of producing tunable 2D flow fields for *in situ* scattering experiments [Fig. 5(d)]. The ratio of the flow rates ( $Q_{1}$ and $Q_{2}$) in each channel determines the flow type, −1<Λ<1, where Λ=1, Λ=0, and Λ=−1 correspond to pure extension, simple shear, and solid-body rotational flows, respectively. This device enables the generation of different types of deformations, including mixed flows, and a vast number of flow histories. The authors developed an analysis based on the characterization of the fluid anisotropy at two different length scales probed via scanning-SAXS.^37^ The extent of fluid anisotropy is captured at a length scale that provides information regarding the particle alignment ( $\alpha_{\mathrm{high}-q}$) and a length scale that contains information on both the particle alignment and the spatial distribution of the neighboring particles ( $\alpha_{\mathrm{low}-q}$), see Fig. 5(e). The ratio $\alpha_{\mathrm{low}-q}/\alpha_{\mathrm{high}-q}$ yields a model-independent index that captures whether the orientational order is dominated by repulsive ( $\alpha_{\mathrm{low}-q}/\alpha_{\mathrm{high}-q}<1$) or attractive interactions ( $\alpha_{\mathrm{low}-q}/\alpha_{\mathrm{high}-q}>1$) between particles. Using this approach, Corona *et al.*^37^ could isolate the effect of flow type on interparticle interactions for two types of naturally derived CR, namely, CNC and relatively more flexible CNF. This approach is useful to understand whether interparticle attractions or repulsions are at play during the flow-induced alignment of the CR. For CNF and CNC dispersions investigated, Corona *et al.*^37^ could distinguish $\alpha_{\mathrm{low}-q}/\alpha_{\mathrm{high}-q}>1$ and $\alpha_{\mathrm{low}-q}/\alpha_{\mathrm{high}-q}<1$ for the CNF and CNC, respectively [Fig. 5(e)]. Additionally, the authors revealed the flow type-dependent alignment of CNC, with the extensional flow being more effective than the simple shear flow at inducing the CNC alignment [Fig. 5(e)]. As such, for the same magnitude of the deformation rate (10 $s^{-1}$), greater values of $\alpha_{\mathrm{low}-q}$ and $\alpha_{\mathrm{high}-q}$ could be achieved under extension-dominated flows [red data points in Fig. 5(e)] compared to shear-dominated flows [green data points in Fig. 5(e)]. Contrarily, the CNF did not display specific correlation between the flow type and the extent of alignment, a feature attributed to pronounced interparticle interactions. In our previous study,^9^ we reported on the extensional flows being more effective at inducing the alignment of dilute CNC dispersion when compared to shearing flows, in line with the finding of Corona *et al.*^37^ and numerical simulations of rigid rods in the dilute (at $\nu l_{c}^{3}=0.05$) and semi-dilute (at $\nu l_{c}^{3}=1.1$) regime.^73^ Recent experiments on two worm-like block copolymers with distinct flexibility ( $l_{c}/l_{p}\approx 5$ and $l_{c}/l_{p}\approx 14$) suggest that the difference between shearing and extensional deformation rates required to induce CR alignment increases with increasing particle flexibility.^33^ Specifically, the ratio of critical extensional and shear rates for the onset of alignment (denoted by the superscript ${}^{*}$) $\left|{\dot{\varepsilon }}^{*}|/|{\dot{\gamma }}^{*}\right|$ appears to increase with $l_{c}/l_{p}$. In this study, we retrieved $\left|{\dot{\varepsilon }}^{*}|/|{\dot{\gamma }}^{*}\right|$ as a function of only two distinct $l_{c}/l_{p}$ values, restricting us from describing a clear trend, yet opening an interesting avenue for further investigations.

## IV. CONCLUSIONS AND OUTLOOK

We have discussed typical microfluidic-based experiments that can be used to probe the structural dynamics of CR under controlled shear and extensional flows. We addressed potential areas of interest for future works, focusing on naturally derived CR in shear and extensional flows. Specifically, we pinpointed the requirement of additional experimental data regarding the CR microstructure-bulk property (particle alignment-rheology) relationship. For instance, it is valuable to understand the dependence of the shear viscosity, $\eta (\dot{\gamma })$, on the CR alignment for CR with varying contour length, flexibility, and for different CR concentrations. This type of study could elucidate the extent of generality of the observed exponential decay of $\eta (\dot{\gamma })$ with the extent of CR alignment. Additionally, it would be of fundamental interest to study the CR alignment-rheology relationship in extensional flows. As explained in Sec. III, FIB and SAS can probe the CR alignment in extensional flows with relative ease; however, probing the extensional viscosity of weakly elastic CR dispersion remains challenging.

Based on our recent work on CR and the literature on soluble polymers in extensional flows, we highlighted the correlation between CR flexibility and CR propensity to align in an extensional-dominated flow. Although a qualitative link is somewhat clear, a quantitative description of this phenomenon is missing and could potentially provide a method to obtain a measure of particle flexibility using microfluidic tools. Overall, more experimental and theoretical research and a combination of the two are needed to obtain a generalized understanding of the dynamics of naturally derived CR under flow.

## Data Availability

The data that support the findings of this study are available from the corresponding author upon reasonable request.
